# Editorial on the Special Issue “Comorbidities in Chronic Kidney Disease”

**DOI:** 10.3390/toxins12060384

**Published:** 2020-06-11

**Authors:** Heidi Noels, Joachim Jankowski

**Affiliations:** 1 Institute of Molecular Cardiovascular Research (IMCAR), RWTH Aachen, University Hospital Aachen, 52074 Aachen, Germany; 2 Department of Pathology, Cardiovascular Research Institute Maastricht, Maastricht University Medical Centre, 6200 Maastricht, The Netherlands

**Keywords:** chronic kidney disease, cardiovascular, CKD–MBD, comorbidity, inflammation, fibrosis, calcification, senescence, uremic toxin

With a mean worldwide prevalence of 13.4% [1], chronic kidney disease (CKD) imposes a massive health burden on our society. In addition to a reduced kidney function, patients with CKD suffer increasingly from **cardiovascular disease** (CVD) [2,3,4], with CVD accounting for around half of the deaths of patients in CKD stages 4–5 [3]. In fact, CKD has been identified as an independent risk factor for CVD [5], but therapeutic options are highly inadequate. In addition to traditional cardiovascular risk factors, CKD-specific pathological mechanisms are expected to contribute to increased cardiovascular risk in this patient group, especially with progressing CKD [6,7,8]. However, detailed insights into the underlying pathophysiology of CKD-driven CVD largely remain to be unveiled [9,10].

**Inflammation** and **fibrosis** are increased in CKD patients [11,12,13], and the Chronic Renal Insufficiency Cohort (CRIC) study recently revealed that inflammation biomarkers are independently associated with atherosclerotic cardiovascular events and death in CKD patients [14]. Moreover, **vascular calcification** is highly prevalent in CKD patients, increases with declining kidney function [15] and is associated with increased risk of cardiovascular events and death in CKD [16,17,18,19]. As one aspect, **uremic retention solutes**, also referred to as uremic toxins, accumulate in the circulation of CKD patients due to a failing kidney filtration function [20]. Many of these solutes have been associated with pathophysiological effects, including inflammation, oxidative stress and calcification. As a consequence, they are expected to contribute to increased cardiovascular risk in CKD patients [21].

Furthermore, patients with CKD often present with enhanced bone demineralization along with extraosseous calcification, a condition clinically referred to as **CKD-mineral and bone disorder** (CKD–MBD). CKD–MBD highly coincides with increased vascular calcification and correlates with cardiovascular events, underlining the importance of identifying and characterizing CKD–MBD biomarkers as well as mediators of this pathological bone–vascular axis [22]. Moreover, patients with CKD present **disturbances of gut microbiota** [23], which too are expected to contribute to both reduced bone and cardiovascular health in CKD patients.

This Special Issue aims to provide insights into comorbidities in CKD patients with a main focus on increased cardiovascular risk and summarizes the current knowledge of underlying pathophysiological mechanisms.

## 1. Increased Cardiovascular Risk in CKD

Patients with CKD have an increased risk of atherosclerosis-related cardiovascular events, such as myocardial infarction and stroke [3,24]. However, with declining renal function, CKD patients are also becoming more prone to non-atherosclerotic cardiovascular events. Underlying cardiac remodeling involves left-ventricular hypertrophy, fibrosis and capillary rarefaction, and is often referred to as uremic cardiomyopathy. In this Special Issue, Kaesler et al. [25] provide detailed insights into cardiac remodeling in CKD and provide an update on the current knowledge of the cellular and molecular mechanisms of pathophysiological kidney–heart crosstalk in CKD patients. This includes alterations in relation to phosphate homeostasis, uremic toxins, growth factors, metabolic and oxidative stress, inflammation as well as fibrosis. Moreover, an overview of current mouse models to study cardiac remodeling in CKD is provided and potential therapeutic targets are being discussed in the context of current knowledge. This underlines the urgent need to further invest in closely studying the pathological crosstalk between kidney and heart in order to guide the development of effective therapies.

## 2. Inflammation and Vascular Calcification in CKD Impact on Cardiovascular Health

Chronic low-grade inflammation is a hallmark of CKD and is closely associated with cellular senescence and accelerated ageing. In this Special Issue, Ebert et al. [26] elaborate on this so-called “inflammageing” in CKD. They address the phenotype of inflammation and premature ageing in CKD patients as well as their mutual activation. Underlying cellular and molecular mechanisms are summarized with a focus on cellular senescence, uremic toxins, the phosphate–FGF23–Klotho axis and the CKD-mediated downregulation of NRF2 as a key transcription factor protecting from mitochondrial dysfunction and oxidative stress. Promising therapeutic candidates to reduce inflammageing in CKD are discussed.

Uremia and uremic toxins not only trigger inflammation, but also accelerate vascular calcification in CKD. This was recently shown for the protein-bound uremic toxins indoxyl sulfate and p-cresyl sulfate, with underlying cellular and molecular mechanisms discussed in detail in this Special Issue by Opdebeeck et al. [27]. Along this line, Lai. et al. [28] reveals within this Special Issue that p-cresyl sulfate is a predictor of arterial stiffness in patients on hemodialysis, with arterial stiffness known to be associated with increased cardiovascular risk and mortality in CKD patients [29,30].

Although vascular calcification has been associated with increased cardiovascular risk, there are currently no therapies available that adequately tackle this pathological axis. This is being discussed by Himmelsbach et al. [31]: a detailed overview of new potential therapeutic strategies to reduce cardiovascular calcification in CKD is provided, covering findings from in vitro molecular studies and animal models to observational and interventional studies in CKD patients.

## 3. CKD–MBD as a Major Complication in CKD Affects Cardiovascular Health

Vascular calcification and bone demineralization often coincide in CKD patients, which is often referred to as the bone–vascular axis or “calcification paradox”. In this Special Issue, Rroji et al. [32] discuss the pathophysiology of CKD–MBD and its association with increased cardiovascular risk. Insights are provided for how vitamin D deficiency, secondary hyperparathyroidism and hyperphosphatemia, as classical CKD-MBD biomarkers, could impact cardiac remodeling in uremic cardiomyopathy. Furthermore, accumulating data supporting a role for FGF23, Klotho-deficiency and sclerostin as new CKD-MBD biomarkers in early cardiovascular risk assessment are discussed in detail, and a role beyond biomarker function and as mediators of cardiovascular risk in CKD is being elaborated on.

Muñoz-Castañeda et al. [33] further elaborate on the FGF23–Klotho axis, its regulation by the Wnt/β-catenin signaling pathway and vice versa. Starting from their deregulation in CKD, the impact of these axes on pathophysiological processes underlying CKD progression as well as cardiovascular disease and bone disorders are being discussed in detail.

Duque et al. [34] specifically focused on secondary hyperparathyroidism as a complication of CKD, with its causes as well as its impact on the bone–vascular axis being discussed. In extension, current literature in relation to a potential impact of secondary hyperparathyroidism on CKD progression, cardiac remodeling, muscle weakness as well as glucose metabolism is summarized.

Furthermore, with CKD patients presenting with gut dysbiosis, Evenepoel et al. [35] provide detailed insights into the increasing evidence that CKD-associated gut dysbiosis contributes to the pathophysiology of the bone-vascular axis. This may include pathophysiological processes such as increased exposure to protein fermentation metabolites, decreased systemic levels of specific short-chain fatty acids by reduced carbohydrate fermentation, vitamin K deficiency as well as a leaky gut triggering a pro-inflammatory environment in CKD.

## 4. Chronodisruption in CKD: Implications for Kidney and Cardiac Health?

Finally, the concept of chronodisruption as a chronic disturbance of circadian rhythms with a negative impact on health is being discussed in the context of CKD. **Carriazo et al**. [36] review current evidence for chronodisruption in CKD as well as its potential impact on kidney and cardiac pathology. Among others, diet, inflammatory factors and uremic toxins are being discussed as potential chronodisrupters in CKD, and the main challenges and open questions regarding the underlying mechanisms, implications for kidney–cardiac health, as well as therapeutic opportunities are summarized.

## 5. Conclusions

Altogether, this Special Issue summarizes current knowledge on the pathophysiological mechanisms underlying the development of comorbidities in CKD, with a main focus on CVD. This reveals the urgent need to further invest efforts in uncovering CKD-specific cardiovascular pathological mechanisms and mediators of disease in order to pave the way for new therapeutic strategies, tailored specifically to the CKD patient.

## References

[1] Hill N.R., Fatoba S.T., Oke J.L., Hirst J.A., O’Callaghan C.A., Lasserson D.S., Hobbs F.R. (2016). Global prevalence of chronic kidney disease—A systematic review and meta-analysis. PLoS ONE.

[2] Gansevoort R., Correa-Rotter R., Hemmelgarn B., Jafar T.H., Heerspink H.J.L., Mann J.F., Matsushita K., Wen C.P. (2013). Chronic kidney disease and cardiovascular risk: Epidemiology, mechanisms, and prevention. Lancet (Lond. Engl.).

[3] Thompson S., James M., Wiebe N., Hemmelgarn B., Manns B., Klarenbach S., Tonelli M. (2015). Cause of death in patients with reduced kidney function. J. Am. Soc. Nephrol..

[4] Valholder R., Massy Z., Argiles A., Spasovski G., Verbeke F., Lameire N. (2005). Chronic kidney disease as cause of cardiovascular morbidity and mortality. Nephrol. Dial. Transplant. Off. Publ. Eur. Dial. Transpl. Assoc. Eur. Ren. Assoc..

[5] Tonelli M., Muntner P., Lloyd A., Manns B., Klarenbach S., Pannu N., James M.T., Hemmelgarn B.R. (2012). Risk of coronary events in people with chronic kidney disease compared with those with diabetes: A population-level cohort study. Lancet (Lond. Engl.).

[6] Fleischmann E.H., Bower J.D., Salahudeen A.K. (2001). Are conventional cardiovascular risk factors predictive of two-year mortality in hemodialysis patients?. Clin. Nephrol..

[7] Alani H., Tamimi A., Tamimi N. (2014). Cardiovascular co-morbidity in chronic kidney disease: Current knowledge and future research needs. World J. Nephrol..

[8] Ortiz A., Covic A., Fliser D., Fouque D., Goldsmith D., Kanbay M., Mallamaci F., Massy Z.A., Rossignol P., Vanholder R. (2014). Epidemiology, contributors to, and clinical trials of mortality risk in chronic kidney failure. Lancet (Lond. Engl.).

[9] Marx N., Noels H., Jankowski J., Floege J., Fliser D., Böhm M. (2018). Mechanisms of cardiovascular complications in chronic kidney disease: Research focus of the transregional research consortium SFB TRR219 of the University Hospital Aachen (RWTH) and the Saarland University. Clin. Res. Cardiol. Off. J. Ger. Card. Soc..

[10] Noels H., Boor P., Goettsch C., Hohl M., Jahnen-Dechent W., Jankowski V., Kindermann I., Kramann R., Lehrke M., Linz D. (2018). The new SFB/TRR219 research centre. Eur. Heart J..

[11] Zoccali C., Vanholder R., Massy Z.A., Ortiz A., Sarafidis P., Dekker F.J., Fliser D., Fouque D., Heine G.H., on behalf of the European Renal and Cardiovascular Medicine (EURECA-m) Working Group of the European Renal Association—European Dialysis Transplantation Association (ERA-EDTA) (2017). The systemic nature of CKD. Nat. Rev. Nephrol..

[12] Stenvinkel P., Wanner C., Metzger T., Heimbürger O., Mallamaci F., Tripepi G., Malatino L., Zoccali C. (2002). Inflammation and outcome in end-stage renal failure: Does female gender constitute a survival advantage?. Kidney Int..

[13] Ruiz-Ortega M., Rayego-Mateos S., Lamas S., Ortiz A., Rodrigues-Diez R.R. (2020). Targeting the progression of chronic kidney disease. Nat. Rev. Nephrol..

[14] Amdur R.L., Feldman H.I., Dominic E.A., Anderson A.H., Beddhu S., Rahman M., Wolf M., Reilly M., Ojo A., Townsend R.R. (2019). Use of measures of inflammation and kidney function for prediction of atherosclerotic vascular disease events and death in patients with CKD: Findings from the CRIC study. Am. J. Kidney Dis. Off. J. Natl. Kidney Found..

[15] Budoff M.J., Rader D.J., Reilly M.P., Mohler E.R., Lash J., Yang W., Rosen L., Glenn M., Teal V., Feldman H.I. (2011). Relationship of estimated GFR and coronary artery calcification in the CRIC (Chronic Renal Insufficiency Cohort) study. Am. J. Kidney Dis. Off. J. Natl. Kidney Found..

[16] Raggi P. (2017). Cardiovascular disease: Coronary artery calcification predicts risk of CVD in patients with CKD. Nat. Rev. Nephrol..

[17] Schlieper G., Schurgers L., Brandenburg V., Reutelingsperger C., Floege J. (2016). Vascular calcification in chronic kidney disease: An update. Nephrol. Dial. Transpl..

[18] Okuno S., Ishimura E., Kitatani K., Fujino Y., Kohno K., Maeno Y., Maekawa K., Yamakawa T., Imanishi Y., Inaba M. (2017). Presence of abdominal aortic calcification is significantly associated with all-cause and cardiovascular mortality in maintenance hemodialysis patients. Am. J. Kidney Dis. Off. J. Natl. Kidney Found..

[19] Claes K., Heye S., Bammens B., Kuypers D., Meijers B.K., Naesens M., Vanrenterghem Y., Evenepoel P. (2013). Aortic calcifications and arterial stiffness as predictors of cardiovascular events in incident renal transplant recipients. Transpl. Int. Off. J. Eur. Soc. Organ Transplant..

[20] Vanholder R., de Smet R., Glorieux G., Argilés A., Baurmeister U., Brunet P., Clark W., Cohen G., De Deyn P.P., for the European Uremic Toxin Work Group (EUTox) (2003). Review on uremic toxins: Classification, concentration, and interindividual variability. Kidney Int..

[21] Lekawanvijit S. (2018). Cardiotoxicity of uremic toxins: A driver of cardiorenal syndrome. Toxins.

[22] Covic A., Vervloet M., Massy Z.A., Torres P.U., Goldsmith D., Brandenburg V.M., Mazzaferro S., Evenepoel P., Bover J., Apetrii M. (2018). Bone and mineral disorders in chronic kidney disease: Implications for cardiovascular health and ageing in the general population. Lancet Diabetes Endocrinol..

[23] Vaziri N., Wong J., Pahl M., Piceno Y., Yuan J., DeSantis T.Z., Ni Z., Nguyen T.-H., Andersen G. (2013). Chronic kidney disease alters intestinal microbial flora. Kidney Int..

[24] Lindner A., Charra B., Sherrard D.J., Scribner B.H. (1974). Accelerated atherosclerosis in prolonged maintenance hemodialysis. N. Engl. J. Med..

[25] Kaesler N., Babler A., Floege J., Kramann R. (2020). Cardiac remodeling in chronic kidney disease. Toxins.

[26] Ebert T., Pawelzik S.-C., Witasp A., Arefin S., Hobson S., Kublickiene K., Shiels P., Bäck M., Stenvinkel P. (2020). Inflammation and premature ageing in chronic kidney disease. Toxins.

[27] Opdebeeck B., D’Haese P.C., Verhulst A. (2020). Molecular and cellular mechanisms that induce arterial calcification by indoxyl sulfate and P-cresyl sulfate. Toxins.

[28] Lai Y.-H., Wang C.-H., Kuo C.-H., Lin Y.-L., Tsai J.-P., Hsu B.-G. (2019). Serum P-cresyl sulfate is a predictor of central arterial stiffness in patients on maintenance hemodialysis. Toxins.

[29] Blacher J., Guerin A., Pannier B., Marchais S.J., Safar M.E., London G.M. (1999). Impact of aortic stiffness on survival in end-stage renal disease. Circulation.

[30] Vlachopoulos C., Aznaouridis K., Stefanadis C. (2010). Prediction of cardiovascular events and all-cause mortality with arterial stiffness: A systematic review and meta-analysis. J. Am. Coll. Cardiol..

[31] Himmelsbach A., Ciliox C., Goettsch C. (2020). Cardiovascular calcification in chronic kidney disease-therapeutic opportunities. Toxins.

[32] Rroji M., Figurek A., Spasovski G. (2020). Should we consider the cardiovascular system while evaluating CKD-MBD?. Toxins.

[33] Muñoz-Castañeda J.R., Rodelo-Haad C., De Mier M.V.P.-R., Martin-Malo A., Santamaria R., Rodríguez M. (2020). Klotho/FGF23 and wnt signaling as important players in the comorbidities associated with chronic kidney disease. Toxins.

[34] Duque E.J., Elias R.M., Moysés R.M.A. (2020). Parathyroid hormone: A uremic toxin. Toxins.

[35] Evenepoel P., Dejongh S., Verbeke K., Meijers B. (2020). The role of gut dysbiosis in the bone-vascular axis in chronic kidney disease. Toxins.

[36] Carriazo S., Ramos A., Sanz A., Sanchez-Niño M.D., Kanbay M., Ortiz A. (2020). Chronodisruption: A poorly recognized feature of CKD. Toxins.

